# Multifloroside Suppressing Proliferation and Colony Formation, Inducing S Cell Cycle Arrest, ROS Production, and Increasing MMP in Human Epidermoid Carcinoma Cell Lines A431

**DOI:** 10.3390/molecules25010007

**Published:** 2019-12-18

**Authors:** Xin Zhang, Yamei Li, Zhengping Feng, Yaling Zhang, Ye Gong, Huanhuan Song, Xiaoli Ding, Yaping Yan

**Affiliations:** 1 National Engineering Laboratory for Resource Development of Endangered Crude Drugs in Northwest of China; College of Life Science, Shaanxi Normal University, Xi’an 710062, China; zhang1xin@126.com (X.Z.); yummylee@snnu.edu.cn (Y.L.); fengzhengping@snnu.edu.cn (Z.F.); gongye@snnu.edu.cn (Y.G.); 15667085859@163.com (H.S.); dingding@snnu.edu.cn (X.D.); 2 Key Laboratory of the Ministry of Education for Medicinal Resources and Natural Pharmaceutical Chemistry, College of Life Science, Shaanxi Normal University, Xi’an 710062, China

**Keywords:** anti-cancer activities, multifloroside, 10-oxyderivatives of oleoside secoiridoids, structure-activity relationship, flow cytometry

## Abstract

Multifloroside (**4**), together with 10-hydroxyoleoside 11-methyl ester (**1**), 10-hydroxyoleoside dimethyl ester (**2**), and 10-hydroxyligustroside (**3**), are all secoiridoids, which are naturally occurring compounds that possess a wide range of biological and pharmacological activities. However, the anti-cancer activity of **1**–**4** has not been evaluated yet. The objective of this work was to study the anti-cancer activities of **1**–**4** in the human epidermoid carcinoma cell lines A431 and the human non-small cell lung cancer (NSCLC) cell lines A549. The results indicate that **1**–**4** differ in potency in their ability to inhibit the proliferation of human A431 and A549 cells, and multifloroside (**4**) display the highest inhibitory activity against A431 cells. The structure-activity relationships suggest that the *o*-hydroxy-*p*-hydroxy-phenylethyl group may contribute to the anti-cancer activity against A431 cells. Multifloroside treatment can also inhibit cell colony formation, arrest the cell cycle in the S-phase, increase the levels of reactive-oxygen-species (ROS), and mitochondrial membrane potential (MMP), but it did not significantly induce cell apoptosis at low concentrations. The findings indicated that multifloroside (**4**) has the tendency to show selective anti-cancer effects in A431 cells, along with suppressing the colony formation, inducing S cell cycle arrest, ROS production, and increasing MMP.

## 1. Introduction

Cancer is currently the second leading cause of death globally [1,2,3]. While great strides have been made in the treatment of cancer in recent decades, we still face many open challenges to existing cancer therapies, such as adaptive resistance [4]. Throughout the development of human society, the importance of natural products [4,5,6,7,8,9] and their derivatives [10,11,12] in cancer treatment or prevention is supported by abundant evidence from cancer research [3,13,14], and give hopes to cancer patients.

**Figure 1:**
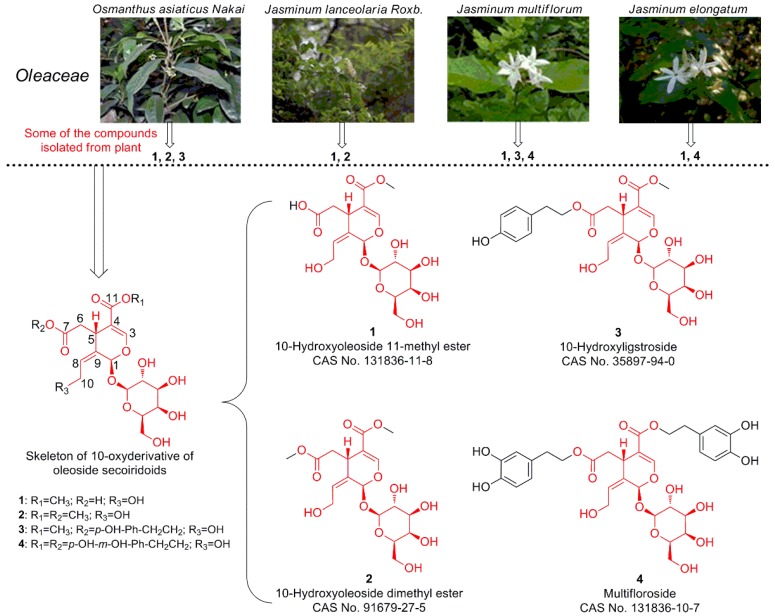
Chemical structures of four 10-oxyderivative of oleoside secoiridoids (**1**–**4**) isolated from plants in the Oleaceae family. The images of the *Oleaceae* plants were downloaded from the Chinese Field Herbarium website (http://www.cfh.ac.cn/default.html).

The *Oleaceae* is a botanical family of woody dicotyledonous plants that are important in daily lives of many people due to their broad economic, food, and medicinal values. As previously reported, a total of 232 secoiridoids (glycosides, aglycones, derivatives, and dimers) have been isolated from species in the *Oleaceae*. Multifloroside (**4**), together with 10-hydroxyoleoside 11-methyl ester (**1**), 10-hydroxyoleoside dimethyl ester (**2**), and 10-hydroxyligustroside (**3**), are 10-oxyderivatives of oleoside secoiridoid (Figure 1), and have been isolated from many *Oleaceae* plants, such as *Osmanthus asiaticus Nakai* [15], *Jasminum lanceolarium* Roxb [16,17], *Jasminum multiflorum* extract [18], and *Jasminum elongatum* (Bergius) Willd [19] (Figure 1). These four 10-oxyderivatives of oleoside secoiridoids (**1**–**4**) are similar in structure, with a hydroxyl substituent at 10 position, one of substituents, such as hydroxyl, methyl, *p*-hydroxy-phenylethyl, or *o*-hydroxy-*p*-hydroxy-phenylethyl, at 7 position, and one of substituents, such as methyl or *o*-hydroxy-*p*-hydroxy-phenylethyl, at 11 position.

No previous anti-cancer studies on **1**–**4** have been reported. Therefore, the study was basically aimed at helping us understand in vitro anti-cancer effect of **1**–**4** against the human epidermoid carcinoma cell lines A431 and the non-small cell lung cancer (NSCLC) cell lines A549. The structure-activity relationships (SAR) and their effect on cell colony formation, apoptosis, cell-cycle distribution, intracellular reactive-oxygen-species (ROS) generation, and the mitochondrial membrane potential (MMP) were also demonstrated in the present study.

## 2. Results

### 2.1. Anti-Proliferative Activity of ***1**–**4*** In Vitro

**Figure 2:**
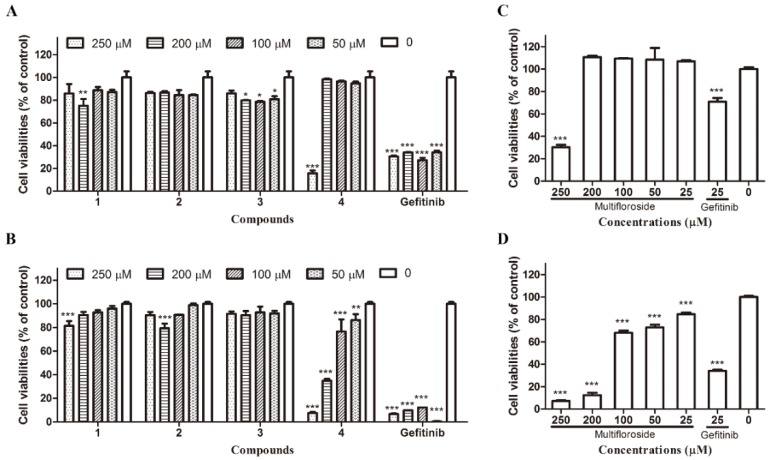
Anti-proliferative activity of compounds in two human cancer cell lines (A549 and A431) as determined by the MTT assay. (**A**) **1**–**4** against A549 cells, (**B**) **1**–**4** against A431 cells, (**C**) Multifloroside (**4**) against A549 cells, (**D**) Multifloroside (**4**) against A431 cells. All results are shown as the mean ± SEM (*n* = 3). * *p* < 0.05, ** *p* < 0.01, and *** *p* < 0.001 indicate significant differences compared with the control.

The 3-(4,5-dimethylthiazol-2-yl)-2,5-diphenyltetrazolium bromide (MTT) assay [20,21] was used to evaluated the anti-proliferative activities of **1**–**4** against the human epidermoid carcinoma cell lines A431 and human NSCLC cell lines A549. Cells were cultured with indicated concentrations (250, 200, 100, and 25 μM) of **1**–**4** or the reference compound gefitinib (an epidermal growth factor receptor inhibitor) for 72 h, and living cells were detected by MTT assay. The results are shown in Figure 2. When against A549 cells, compared with the control cells, significant growth inhibitor effect could be observed when cells were treated with 200 μM of **1**, 200, 100, and 50 μM of **3**, 250 μM of **4**, and 250, 200, 100, and 50 μM of gefitinib (Figure 2A). When against A431 cells, compared with the control cells, significant growth inhibitor effect could be observed when cells were treated by 250 μM of **1**, 200 μM of **2**, and 250, 200, 100, and 50 μM of **4** and gefitinib (Figure 2B). The results are further shown in Figure 2C and D, when A549 cells were treated with 250 μM of **4** (multifloroside) or 25 μM of gefitinib, cell viabilities decreased markedly to 30.30% and 70.85% compared with the control group, respectively (*p* < 0.001), when A431 cells were treated with 250, 200, 100, 50, and 25 μM of **4** (multifloroside) or 25 μM of gefitinib, cell viabilities decreased markedly to 7.21%, 12.44%, 70.29%, 75.87%, 84.62%, and 34.02% compared with the control group, respectively (*p* < 0.001), and the inhibitory effect was concentration-dependent. The above results suggest that **1**–**4** possess different anti-proliferative activities against A549 and A431 cells, and **4** (multifloroside) is the most potent agent against A431 cells.

### 2.2. The Structure-Activity Relationships (SAR)

The structure-activity relationships were analyzed basing on the MTT results, and we found that, in the core structure of 10-oxyderivatives of oleoside secoiridoids, **1**–**4** all had a hydroxyl substituent at the 10 position and only differed at the 7 and 11 positions. **1** had a hydroxyl group at the 7 position and a methyl group at the 11 position, **2** had methyl groups at the 7 and 11 positions, and **3** had a *p*-hydroxy-phenylethyl group at the 7 position and a methyl group at the 11 position, while **4** (multifloroside) had an *o*-hydroxy-*p*-hydroxy-phenylethyl group at both the 7 and 11 positions (Figure 1), which indicated that the *o*-hydroxy-*p*-hydroxy-phenylethyl group may contribute to the anti-proliferative activity of multifloroside against A431 cells.

### 2.3. Multifloroside Inhibits Tumor Cell Colony Formation

**Figure 3:**
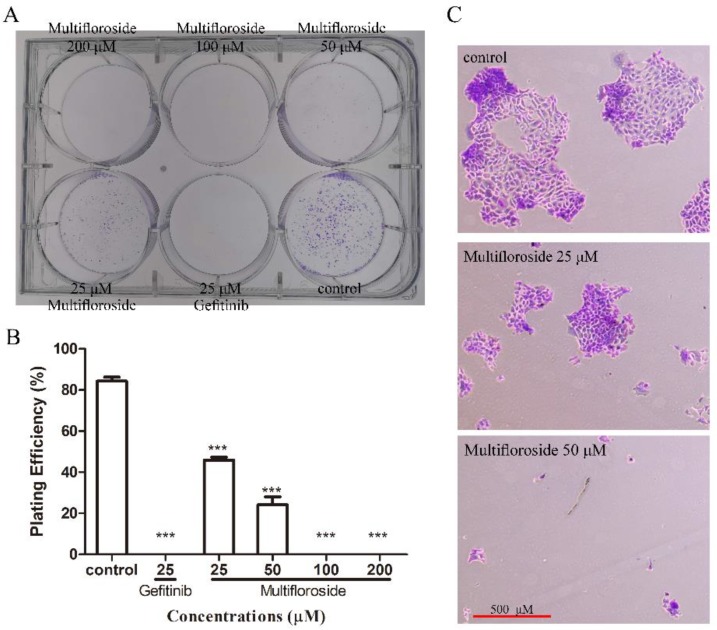
Colony formation of A431 cells inhibited by multifloroside. (**A**) A431 cells were incubated with the indicated concentrations of multifloroside or gefitinib and fixed with 4% paraformaldehyde and stained with 0.2% crystal violet 12 days after cell treatment. (**B**) Bar chart showing the decrease in the number of colonies after incubation with multifloroside. (**C**) Micrographs showing differences between the cell colonies. Images were taken of stained single colonies observed under a microscope. A single colony was defined to be an aggregate of >50 cells. Data are shown as mean ± SEM (*n* = 3), *** *p* < 0.001 indicates a significant difference compared with the control.

Encouraged by the above results, we further investigated the bioactivity of multifloroside. First, the inhibitory effect of multifloroside on the proliferative ability of A431 cells was determined via colony formation assay. Herein, A431 cells were seeded into a 6-well cell culture plate at a density of 400 cells/well and treated with multifloroside at several concentrations (200, 100, 50, and 25 µM), gefitinib (25 µM) or 0.5% DMSO for 12 days, and then stained with crystal violet. As shown in Figure 3, multifloroside resulted in a significant suppression of cell colony formation. When the cells were incubated with multifloroside (200 and 100 µM) or gefitinib (25 µM), colony formation were completely suppressed. In addition, the cells incubated with multifloroside (50 and 25 µM) formed fewer and smaller colonies in a concentration-dependent manner compared to the control group. There were 337 viable colonies when A431 cells were treated with 0.5% DMSO, but only 183 and 76 colonies were observed when the cells were exposed to multifloroside at concentrations of 50 and 25 µM, respectively. Therefore, the plating efficiency (PE) showed a significant decrease compared to the control (*p* < 0.001). The PEs were 84%, 46%, and 24% for the control, and the 25 µM and 50 µM multifloroside treatments, respectively, and were 0 for the other groups. These results demonstrate that multifloroside inhibits the growth of A431 cells and that the inhibitory effect of multifloroside persists for a significant period of time.

### 2.4. Effect of Multifloroside on Cell Apoptosis in A431 Cells

**Figure 4:**
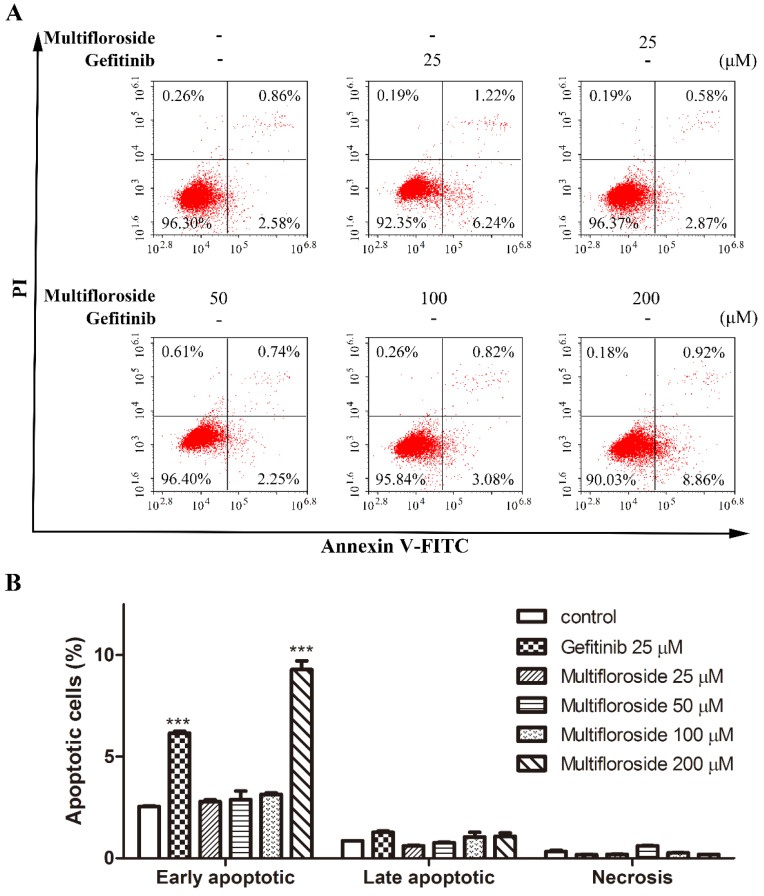
Effect of multifloroside on cell apoptosis in A431 cells. (**A**) Representative histograms of apoptosis in the cells treated with multifloroside for 48 h, (**B**) Percentages of apoptotic cells in each group from (**A**). All values are expressed as mean ± SEM (*n* = 3). *** *p* < 0.001 indicates a significant differences compared with the control at the same group.

After treatment with multifloroside for 48 h, apoptosis of A431 cells was measured by flow cytometry using annexin V-FITC and PI labeling. As shown in Figure 4, 96.30% of A431 cells were in their normal state in the untreated control groups. When cells were treated with multifloroside (200 μM) or gefitinib (25 μM), the numbers of early apoptotic cells were significantly higher than in the control groups (*p* < 0.001). However, there were no significant differences in the numbers of early apoptotic cells when cells exposed to lower concentrations of multifloroside (25, 50 and 100 μM). In addition, there were no significant differences in the numbers of late apoptotic cells and necrotic cells when cells were treated with multifloroside at 25, 50, 100, and 200 μM and gefitinib at 25 μM. These results indicate not only that early apoptosis of A431 cells was not significantly influenced by treatment with low concentrations of multifloroside but also that late apoptosis was not significantly affected by multifloroside at the tested concentrations (25–200 μM).

### 2.5. S-Phase Cell Cycle Arrest Induced by Multifloroside

**Figure 5:**
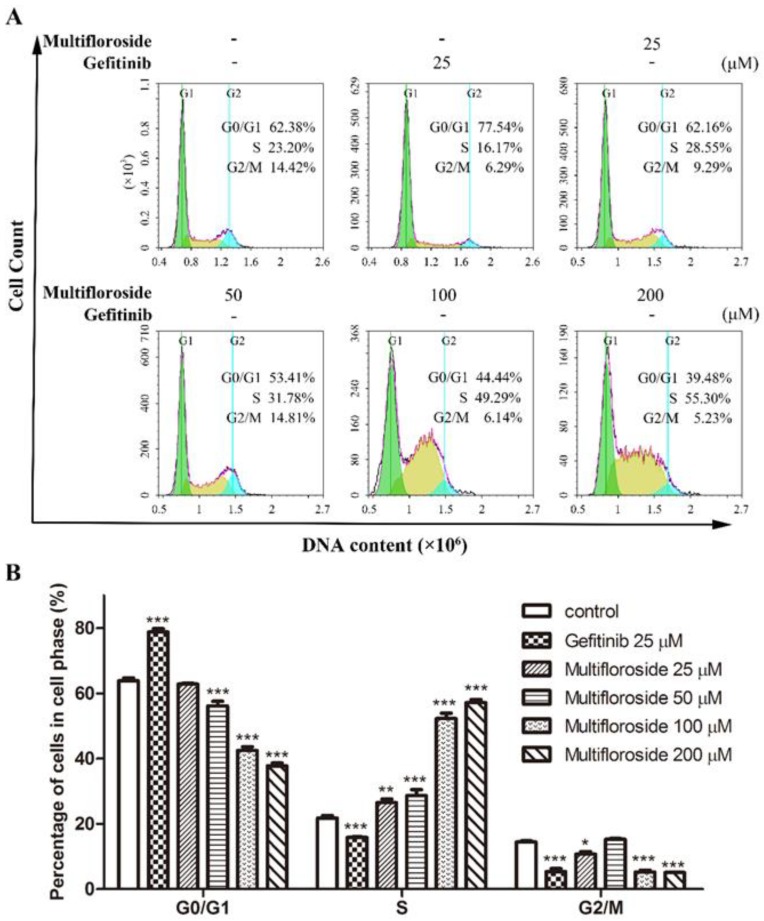
Effect of multifloroside on the cell cycle phase distribution in A431 cells. (**A**) Representative histograms of DNA content in the cells treated with multifloroside for 48 h, and (**B**) percentages of cell populations in the G0/G1, S and G2/M phases from (**A**). All values are expressed as mean ± SEM (*n* = 3). * *p* < 0.05, ** *p* < 0.01, and *** *p* < 0.001 indicate significant differences compared with the control at the same phase.

In order to study the relationship between the anti-proliferative activity of multifloroside and cell cycle arrest, A431 cells were treated with multifloroside or gefitinib for 48 h, after which the cells were stained with PI and examined using flow cytometry. As shown in Figure 5, when cells were treated with multifloroside at four different concentrations (25, 50, 100 and 200 μM), the number of A431 cells in S phase were significantly increased (*p* < 0.001), from 28.55% to 31.78%, 49.29%, and 55.30%, respectively, accompanied by a decrease in the number of cells in G0/G1 and G2/M. In the gefitinib (25 μM) treated cells, the percentage of cells in G0/G1, S, and G2/M were 77.54%, 16.17%, and 6.29%, respectively. These results indicate, unlike gefitinib which arrests the A431 cells in the G0/G1-phase, multifloroside can arrest the cell cycle of A431 cells in the S-phase and showed concentration-dependent activity.

### 2.6. Intracellular ROS Production Induced by Multifloroside

**Figure 6:**
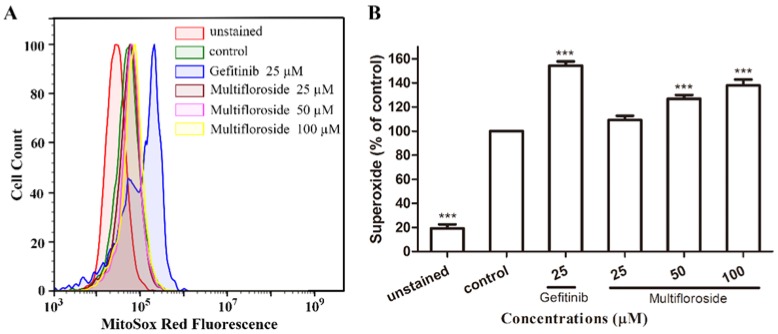
Effect of multifloroside on ROS production. A431 cells were treated with different concentrations of multifloroside for 48 h, then MitoSox red reagent (5 µM) was loaded and the cells were analyzed by flow cytometry for the quantification of multifloroside-induced oxidative stress in A431 cells. The fluorescence intensity of MitoSox Red reagent in cells was obtained by FACS (**A**) and the data was analyzed using GraphPad Prism 5 (**B**). The values are presented as mean ± SEM (*n* = 3). *** *p* < 0.001 indicates a significant differences compared with the control.

Intracellular ROS generation was evaluated via MitoSox red reagent staining and flow cytometry analysis. As shown in Figure 6, treatment with gefitinib (25 μM) significantly increased the fluorescence intensity (*p* < 0.001), and treatment with multifloroside at high concentrations (50 and 100 μM) also significantly increased the fluorescence intensity (*p* < 0.001) in a dose-dependent manner. Treatment with multifloroside at a low concentration (25 μM) also increased the fluorescence intensity, but the difference was not statistically significant (*p* > 0.05). These results indicate that ROS production can be induced by multifloroside at the tested concentrations.

### 2.7. Effect of Multifloroside on the MMP

**Figure 7:**
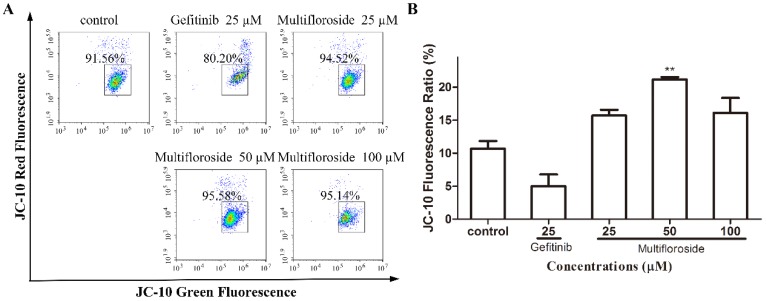
Effect of multifloroside on the MMP of A431 cells. A431 cells were treated with different concentrations of multifloroside for 48 h and analyzed by flow cytometry after JC-10 staining. The fluorescent intensity of JC-10 in cells was obtained by FACS (**A**) and the data was analyzed by GraphPad Prism 5 (**B**). The percentage of cells with JC-10 red fluorescence is indicated. JC-10 fluorescence ratio (%) equals the red/green fluorescence intensity ratio. The values are presented as mean ± SEM (*n* = 3). ** *p* < 0.01 indicates a significant differences compared with the control.

MMP of A431 cells stained with JC-10 was assayed by flow cytometry. As shown in Figure 7, the JC-10 fluorescence ratio (% of J-aggregates with Red Fluorescence divided by the % of J-monomers with Green Fluorescence) decreased but was not significantly different (*p* > 0.05) when cells were treated with gefitinib (25 μM), while the ratios increased when cells were treated with multifloroside (25, 50, and 100 μM). Moreover, the differences in the JC-10 fluorescence ratios were not significant when cells were treated with multifloroside at 25 and 100 μM, and they were significant when cells were treated with 50 μM multifloroside (*p* < 0.01). The above results indicate the MMP did not decrease, but rather increased, with multifloroside treatment

## 3. Discussion

Cancer incidence and mortality are rising rapidly worldwide. Throughout the development of human society, nature has catered to the basic needs of humans, not the least of which is the provision of medicines for the treatment of a wide spectrum of diseases [22,23]. There is growing evidence that specific bioactive compounds present in plants used as spices, fruits, vegetables, and nuts can be effective against some cancers. Secoiridoids, isolated from *Oleaceae* family, which are now widely used in the fields of food and medicine, have been extensively investigated in recent years and have been shown to possess a variety of pharmacological effects [24,25,26,27,28,29], such as anti-diabetic [30,31], anti-oxidant [32,33], anti-inflammatory [34,35,36], immunosuppressive [37], neuroprotective [36,38,39], and anti-cancer [1,40,41,42]. Multifloroside (**4**), together with 10-hydroxyoleoside 11-methyl ester (**1**), 10-hydroxyoleoside dimethyl ester (**2**), and 10-hydroxyligustroside (**3**), are all 10-oxyderivatives of oleoside secoiridoids.

**1**–**3,** together with other eight secoiridoid glycosides, were isolated from the bark of *Osmanthus asiaticus Nakai in 1993* [15]; **2** was isolated from *Jasminum lanceolarium* Roxb in 1997 and 2007 [16,17]; **1** was also isolated from *Jasminum lanceolarium* Roxb in 2007 [16]; **1**, **3**, and **4** were isolated from the water soluble fraction of *Jasminum multiflorum* extract [18]; **1** and **4**, with the other 11 types of iridoid glycosides and 15 other compounds, were identified by UPLC-MS in extracts of *Jasminum elongatum* (Bergius) Willd in 2016 [19]. However, by now, only **1** and **4** have been found to possess coronary dilating and cardiotropic activities [18]. Therefore, we aimed to investigate the in vitro anti-cancer activities of **1**–**4** in this study. Cancer is characterized by uncontrolled cell growth, invasiveness, and formation of metastasis, and the cells of most of malignant tumors are proliferate intensively [14]. Thus, we first evaluated the anti-proliferative activities of **1**–**4** against A431 human epidermoid carcinoma cells and A549 human NSCLC cells. The results showed that **1**–**4** possess different anti-proliferative activities against A549 and A431 cells, and that **4** (multifloroside) had the most potent inhibitory activity against A431 cells (Figure 2). As reported, it appears that the best way to prevent cancer is through rational dietary habits and behaviors, and consumption of sufficient amounts of antioxidants and bioactive plant-derived compounds that demonstrate protective effects against carcinogenesis in pre-clinical and clinical studies [43], **4** (multifloroside) and its derivatives may be the way to prevent epidermoid carcinoma cancer. The SAR were analyzed, and we found that the *o*-hydroxy-*p*-hydroxy-phenylethyl group may contribute to the anti-proliferative activity of multifloroside against A431 cells. In a future study, it may be advantageous to study other oleoside secoiridoids with *o*-hydroxy-*p*-hydroxy-phenylethyl substituents in order to identify the most potent compounds in the *Oleaceae* that can protect against the epidermoid carcinomas.

Second, a colony formation assay was performed to detect the inhibitory effect of multifloroside on the proliferative ability of A431 cells. The assay is now widely used to examine the effect of agents with potential clinical applications [44], and it shows the ability of a cancer cell to produce a viable colony after drug treatment; thus, the results obtained may help to predict the efficacy of agents in vivo [45]. Similarly to other reported plant anti-cancer molecules and their derivatives, such as panaxatriol [46], cycloartenol [47], and oridonin derivatives [48], the number and the size of tumor cell colonies were significantly decreased when A431 cell were treated by 200, 100, 50, and 25 μM multifloroside (Figure 3). These results all illustrate that multifloroside can significantly inhibit the growth of A431 cells, which is consistent with the results of MTT assay.

As reported, most of malignant tumors whose cells proliferate intensively, represent very dynamic structures that create numerous mutations resulting in new tumor cell lines with different genotypes and phenotypes within the tumor mass. In such malignancies, a highly variable sensitivity to therapeutics can be observed, and some of cell lines develop resistance to the treatment [14,49]. Therefore, the biological effects of combining various plant molecules (phytochemicals) with proven cytotoxic effects administered together with conventional therapy to target a markedly wider range of signaling pathways in cancer cells should be superior compared to single compound in cancer treatment and thus may delay the development of drug resistance in cancer [43]. So, further urgent research is needed for the identification of new molecules (including plant-derived compounds) with well-validated anticancer properties within combinational clinical approach in oncology. Further studies were then done to elucidate the mechanism of action of multifloroside on A431 cells. In general, drug-induced apoptosis is one major mechanism of action for the treatment of cancer, and various signaling pathways are involved in the process [50]. However, the apoptotic assay suggested multifloroside at high concentration (200 µM) induced cell apoptosis but that at lower concentrations (100, 50, and 25 µM) could not (Figure 4). Cell cycle regulating is a key method in controlling tumor propagation [50]. As reported, most antitumor compounds inhibit cell proliferation by inducing cell cycle arrest [48]. Cell-cycle analyses indicated the cell-cycle distribution was significantly changed, and was also different from that resulting from gefitinib, where the cells were arrested in the G0/G1-phase, multifloroside-treated cells were arrested cells in the S-phase (Figure 5). Mitochondria are the main source of cellular ROS, which have a double-edged sword role in cytotoxicity in cancer cells [51,52]. Herein, ROS levels were significantly increased after cells were treated with multifloroside at 50 and 100 µM for 48 h (Figure 6). Similarly, the MMP was increased when cells were treated with multifloroside at the tested concentrations compared with the control group (Figure 7). JC-10 forms “J-aggregates” displaying red fluorescence in healthy cells, in contrast, as the membrane potential decreases, JC-10 monomers are generated, resulting in a shift to green fluorescence [52]. These results all suggest that multifloroside may exert its anti-cancer effect through cell-cycle arrest and an increase in the ROS and MMP in A431 cells. The above gives a well-validated anticancer properties for multifloroside and suggests multifloroside may be the potential one in applying to anti-epidermoid carcinoma cancer.

## 4. Methods

### 4.1. Chemicals and Other Reagents

10-hydroxyoleoside 11-methyl ester (**1**, C_17_H_24_O_12_, CAS No. 131836-11-8, MW 420.37, purity 98%), 10-hydroxyoleoside dimethyl ester (**2**, C_18_H_26_O_12_, CAS No. 91679-27-5, MW 434.39, purity 98%), 10-hydroxyligustroside (**3**, C_25_H_32_O_13_, CAS No. 35897-94-0, MW 540.51, purity 97%) and multifloroside (**4**, C_32_H_38_O_16_, CAS No. 131836-10-7, MW 678.64, purity 98%) (Figure 1) were purchased from BioBioPha Co., Ltd. (Kunming, China). MTT was from Merck Millipore. The Annexin V-FITC/PI double staining apoptosis assay kit and Cell Cycle Detection Kit were from KeyGEN Bio Tech (Nanjing, China). MitoSox Red mitochondrial superoxide indicator was obtained from Thermo Fisher (Shanghai, China). The JC-10 Mitochondrial Membrane Potential Assay Kit was from Mybiotech, (Xi’an, China). Dulbecco’s Modified Eagle’s Medium (DMEM) and fetal bovine serum (FBS) were purchased from Gibco (Carlsbad, California, USA). Trypsin, penicillin, streptomycin and l-glutamate were purchased from MP Biomedicals, LLC (Santa Ana, CA, USA). All other chemicals used in this work were analytical grade and were purchased from local commercial suppliers and used without further purification unless otherwise noted.

### 4.2. Chemicals and Other Reagents

Human epidermoid carcinoma cell lines A431 and human NSCLC cell lines A549 were purchased from the Cell Bank of the Chinese Academy of Sciences (Shanghai, China). Cells were maintained in 60 mm cell culture dishes and cultured using DMEM supplemented with 10% FBS, 100 units/mL penicillin, 100 μg/mL streptomycin, and 2 mM l-glutamate at 37 °C in a 5% CO_2_ atmosphere with 95% humidity.

### 4.3. MTT Assay for In Vitro Anti-Proliferative Activity

The effect of compounds on cell proliferation was determined using the MTT assay, which is one of the most versatile and popular assays to assess the rate of cell proliferation caused by drugs and cytotoxic agents [53]. The assays were performed as previously described [52,54,55].

### 4.4. Colony Formation Assay

Cell colony formation assay was performed according to the reference methods [45,48]. Firstly, cells were digested, harvested, and suspended using DMEM medium, and 400 cells/well were seeded into 6-well cell culture plates and incubated with multifloroside (25‒200 μM) or gefitinib (25 μM) for 12 days, until the cells in the control dishes had formed visible colonies. Then the colonies were washed three times with PBS and fixed with 4% paraformaldehyde for 20 min. All cells were stained with 0.2% crystal violet for 20 min. The stained colonies were counted and compared with the control samples. The medium was changed every three days.

### 4.5. Cell Apoptosis Analysis

Following treatment with different concentrations of multifloroside for 48 h, the apoptosis of A431 cells was measured using the Annexin V-FITC/PI apoptosis detection kit according to the manufacturer’s instructions. Apoptosis of the treated cells was then detected by flow cytometry (ACEA NovoCyte™, ACEA Biosciences, San Diego, CA, USA).

### 4.6. Cell Cycle Analysis

Following treatment with different concentrations of multifloroside for 48 h, the distribution of cell cycle was measured using the cell cycle detection kit according to the manufacturer’s instructions. The cell cycle of the treated cells was then detected by flow cytometry to quantify the amount of DNA in each cell (ACEA NovoCyte™, ACEA Biosciences, San Diego, CA, USA).

### 4.7. Detection of Intracellular ROS

Intracellular ROS production was examined using MitoSox Red mitochondrial superoxide indicator according to the manufacturer’s protocol and following our previously described method [52]. In brief, 24 h after plating the cells, the culture medium was replaced with fresh medium containing various concentrations of multifloroside (25‒100 μM) or gefitinib (25 μM). After incubation for 48 h, cells were trypsinized, washed, and stained with MitoSox. Next, cells were washed, centrifuged to remove the supernatant, and resuspended in HBSS. Finally, ROS production in the cells was immediately monitored by flow cytometry (ACEA NovoCyte™, ACEA Biosciences, San Diego, CA, USA).

### 4.8. Mitochondrial Membrane Potential (MMP) Assay

MMP was analyzed using the JC-10 Mitochondrial Membrane Potential Assay Kit according to the manufacturer’s instructions and following our previously described protocol [52]. In brief, 24 h after plating the cells, the culture medium was replaced with fresh medium containing various concentrations of multifloroside (25‒100 μM) or gefitinib (25 μM). After incubation for 48 h, cells were trypsinized, washed, and stained with JC-10 dye. Next, cells were washed, centrifuged to remove the supernatant, and resuspended in PBS. Finally, the red/green fluorescence was detected by flow cytometry (ACEA NovoCyte™, ACEA Biosciences, San Diego, CA, USA).

### 4.9. Statistical Analysis

All measurements were made in triplicate, and all data are expressed as means ± SEM of three independent experiments. The significant differences from the respective control for each experimental group were examined by one-way analysis of variance (ANOVA) using GraphPad Prism 5 software. A value of *p* < 0.05 was considered to be statistically significant, and values of *p* < 0.01 or *p* < 0.001 indicated extremely significant differences.

## 5. Conclusions

In summary, multifloroside (**4**), together with other three 10-oxyderivatives of oleoside secoiridoids (**1**–**3**), were evaluated for their anti-cancer activities in *vitro*. These four compounds differ in their inhibition potency against human cancer cell lines A431 and A549, and interestingly, only multifloroside exhibited the most potent inhibitory activity against A431 cells. Further studies on multifloroside suggest that it can inhibit cell colony formation, arrest the cell cycle in the S-phase, and increase the level of ROS and MMP but cannot exert significant changes in cell apoptosis. These findings can help us understand the structure-activity relationships behind the tumor-inhibitory effect of **1**–**4** against the two tumor cell lines and have important implications for the potential use of multifloroside in the treatment of human epidermoid carcinoma. In a subsequent study, we will identify and design novel derivatives of multifloroside, investigate their activities in vitro and in vivo, and provide additional information on the ability of these compounds to protect against epidermoid carcinoma cancer.
